# Coincident Helminth Infection Modulates Systemic Inflammation and Immune Activation in Active Pulmonary Tuberculosis

**DOI:** 10.1371/journal.pntd.0003289

**Published:** 2014-11-06

**Authors:** Parakkal Jovvian George, Nathella Pavan Kumar, Rathinam Sridhar, Luke E. Hanna, Dina Nair, Vaithilingam V. Banurekha, Thomas B. Nutman, Subash Babu

**Affiliations:** 1 National Institutes of Health—NIRT—International Center for Excellence in Research, Chennai, India; 2 Government Stanley Medical Hospital, Chennai, India; 3 National Institute for Research in Tuberculosis, Chennai, India; 4 Laboratory of Parasitic Diseases, National Institutes of Allergy and Infectious Diseases, National Institutes of Health, Bethesda, Maryland, United States of America; University of California San Diego School of Medicine, United States of America

## Abstract

**Background:**

Helminth infections are known to modulate innate and adaptive immune responses in active and latent tuberculosis (TB). However, the role of helminth infections in modulating responses associated with inflammation and immune activation (reflecting disease activity and/or severity) in TB is not known.

**Methodology:**

We measured markers of inflammation and immune activation in active pulmonary TB individuals (ATB) with co-incidental *Strongyloides stercoralis* (Ss) infection. These included systemic levels of acute phase proteins, matrix metalloproteinases and their endogenous inhibitors and immune activation markers. As a control, we measured the systemic levels of the same molecules in TB-uninfected individuals (NTB) with or without Ss infection.

**Principal Findings:**

Our data confirm that ATB is associated with elevated levels of the various measured molecules when compared to those seen in NTB. Our data also reveal that co-incident Ss infection in ATB individuals is associated with significantly decreased circulating levels of acute phase proteins, matrix metalloproteinases, tissue inhibitors of matrix metalloproteinases as well as the systemic immune activation markers, sCD14 and sCD163. These changes are specific to ATB since they are absent in NTB individuals with Ss infection.

**Conclusions:**

Our data therefore reveal a profound effect of Ss infection on the markers associated with TB disease activity and severity and indicate that co-incidental helminth infections might dampen the severity of TB disease.

## Author Summary

Helminth-induced changes in the immune system are thought to influence the outcome of secondary infections. Approximately 50–100 million people are thought to have infection by worms known as *Strongyloides stercoralis*, while more than 2 billion people worldwide are infected with the bacterium, *Mycobacterium tuberculosis*. Interestingly, there is a great degree of overlap in the geographical spread of both infections. We and others have previously shown that helminth infections induce modulation of innate and adaptive immune responses in tuberculosis. In this study, we examined whether concomitant helminth infection has a secondary effect on systemic markers of disease severity/activity in pulmonary tuberculosis. We show that helminth infection have profound effect on lowering most of the circulating parameters associated with tuberculosis pathology. We therefore, conclude that helminth infections have both beneficial and detrimental effects on the progression of tuberculosis, with the beneficial effect manifest upon development of pathology.

## Introduction

Tuberculosis is a major public health problem worldwide with nearly 10 million new cases occurring each year [1]. Tuberculosis manifests as a disease spectrum ranging from latent infection to overt pulmonary or extra-pulmonary disease. Active TB reflects the progression from latent TB to active symptomatic disease, a progression reflecting the failure to contain the mycobacteria within granulomata [1]. While adaptive immune responses play a major role in the pathogenesis of TB disease, it is also abundantly clear that systemic and local inflammatory and innate parameters significantly influence disease activity and severity [2]. Commonly used markers to estimate TB disease severity and/or activity are factors that are essential for the underlying pathological process of TB disease and usually signify non-specific inflammatory processes that are reflected in increases in acute phase proteins, matrix metalloproteinases and systemic markers of immune activation [3].

Helminth parasites are complex eukaryotic organisms, characterized by their ability to maintain long-standing infections in humans, sometimes lasting decades. Perhaps the most long-lived (because of its autoinfective life cycle), *Strongyloides stercoralis*, the causative agent of strongyloidiasis, infects about 50–100 million people worldwide [4], [5]. Strongyloides (Ss) infection is often clinically asymptomatic due, in large part, to the parasites' ability to manipulate the host immune system and to restrict local and perhaps systemic inflammatory pathology [6], [7]. In addition, this ability to curb host inflammatory pathology has rendered helminths as potentially useful tools to control and ameliorate disease processes that are characterized by exacerbated inflammation, including autoimmune diseases [8]. Helminth infections commonly occur in resource limited parts of the world and share a great degree of overlap in geographic distribution with TB [9]. Recent, epidemiological and experimental evidence provide evidence that helminths (both systemic and intestinal) negatively influence TB infection and disease [9].

Helminth infections are known to modulate adaptive immune responses to TB antigens in both latent and active TB and to affect the clinical progression of disease [10]. However, their effect on immune-mediated pathology in active TB has not, to our knowledge, been examined systematically. Moreover, since Ss is an intestinal helminth with a lung migratory larval stage, it could directly influence TB disease pathology. We therefore hypothesized that the regulatory networks established during chronic Ss infection could potentially modulate the inflammatory processes driven by TB infection and disease. Thus, we examined the systemic levels of a variety of systemic markers, shown previously to reflect disease activity and/or severity in TB. Although, we did not find any effect on Ss infection on mycobacterial burdens, we did observe a profound modulating effect of coincidental helminth infection on most of the major markers felt to influence immune-mediated pathology in active pulmonary TB,

## Materials and Methods

### Ethics statement

All individuals were examined as part of a research protocol approved by Institutional Review Board of the National Institute for Research in Tuberculosis, and informed written consent was obtained from all participants.

### Study population

We studied a group of 69 individuals with active pulmonary TB, 33 of whom were infected with *S. stercoralis* (hereafter ATB+Ss) infection and 36 of whom had active TB alone (ATB) in Tamil Nadu, South India (Table 1). Another set of 46 individuals were used as TB - uninfected controls (hereafter NTB), of whom 23 were infected with *S. stercoralis* (hereafter NTB+Ss) infection. Individuals were recruited from patients and their relatives attending the outpatient clinic at the Stanley Medical Hospital, Chennai. Active pulmonary TB was diagnosed microbiologically on the basis of being at least culture positive for Mtb by solid cultures in LJ medium (some were also sputum smear positive). NTB individuals were asymptomatic with normal chest radiographies and with negative sputum smears, cultures and Quantiferon tests. Ss infection was diagnosed by the presence of IgG antibodies to the 31-kDa recombinant NIE antigen by the Luciferase Immunoprecipitation System Assay, as described previously [11]. All individuals were also negative for filarial infection by filarial antigen tests but stool microscopy for intestinal helminths was not done. All individuals were HIV negative and anti-tuberculous and anthelmintic treatment naive. The two groups of active TB individuals did not differ significantly in bacillary burden (as estimated by smear grades at the time of diagnosis following Ziehl-Nielsen staining).

**Table 1:** Study demographics.

	ATB (n = 36)	ATB+Ss (n = 33)	NTB (n = 23)	NTB+Ss (n = 23)
**Age**	50 (23–70)	45 (19–65)	40 (20–63)	45 (18–61)
**M/F**	30/6	29/4	8/15	10/13
**Smear grade 0/1+/2+/3+**	2/11/8/15	4/11/9/9	Negative	Negative
**NIE LIPS Assay**	Negative	Positive	Negative	Positive

ATB: Active TB; NTB: TB uninfected; Ss: Strongyloides infected; NIE LIPS: Strongyloides NIE antigen based Luciferase Immunoprecipitation.

### Acute-phase proteins

Plasma levels of C-reactive protein (CRP), haptoglobin, serum amyloid A (SAA), and α-2 macroglobulin (α-2M) were measured using the Bioplex (Bio-Rad, Hercules, CA) multiplex ELISA system according to the manufacturer's instructions.

### Matrix metalloproteinases (MMPs) and Tissue inhibitor of metalloproteinases (TIMPs)

Plasma samples were assayed for MMP-1, MMP-7, MMP-8 and MMP-9 using the R&D (Minneapolis, MN) multiplex ELISA system according to the manufacturer's instructions. TIMP-1, TIMP-2, TIMP-3, and TIMP-4 levels were measured using the R&D multiplex ELISA system according to the manufacturer's instructions.

### Immune activation markers

Plasma levels of sCD14 (Bio-Rad), sCD163 (R&D), sPD-1 (R&D) and Platelet derived growth factor (PDGF; Biorad) were measured by ELISA.

### Statistical analysis

Data analyses were performed using GraphPad PRISM (GraphPad Software, Inc., San Diego, CA, USA). Geometric means (GM) were used for measurements of central tendency. Comparisons were made using either the Mann-Whitney U test with Holm's correction for multiple comparisons or the Kruskal-Wallis test with Dunn's correction for multiple comparisons.

## Results

### Elevated circulating levels of acute phase proteins, MMPs, TIMPs and immune activation markers in active pulmonary TB

Acute phase proteins, MMPs, TIMPs and systemic immune activation markers are known to reflect disease activity and/or severity in pulmonary TB [2], [3]. To verify these data in our study group, we measured the circulating levels of acute phase proteins (α-2M, CRP, SAA and Haptoglobin), MMPs (MMP-1, 7, 8, 9), TIMPs (TIMP-1, 2, 3, 4) and immune activation markers (sCD14, sCD163, sPD-1 and PDGF) in ATB and compared them to those in NTB individuals. As shown in Table 2, ATB individuals exhibited significantly higher levels of α-2M, CRP and SAA in comparison to NTB individuals. Similarly, ATB individuals exhibited significantly higher levels of MMP-1, MMP-8 and MMP-9 but not MMP-7 in comparison to ATB individuals (Table 2). In addition, ATB individuals also exhibited significantly higher levels of TIMP-1, TIMP-2 and TIMP-4 but not TIMP-3 in comparison to NTB individuals (Table 2). Finally, as shown in Table 2, ATB individuals also exhibited significantly higher levels of sCD14, sPD-1 and PDGF in comparison to NTB individuals. Our data thus confirms previous reports that acute phase proteins, MMPs, TIMPs and immune activation reflect disease activity and severity in ATB.

**Table 2:** Systemic levels of immune parameters in active TB and TB uninfected individuals.

	ATB	NTB	p value
**α-2-Macroglobulin**	4.007 (3.73–4.66)	0.8537 (0.76–1.04)	<0.0001
**C-Reactive Protein**	6.16 (5.86–6.79)	0.08126 (0.03–0.47)	<0.0001
**Serum Amyloid Protein**	4.31 (4.07–4.86)	0.7393 (0.6–1.08)	<0.0001
**Haptoglobin**	1.286 (1.17–1.60)	1.435 (1.31–2.29)	NS
**MMP-1**	5.269 (5.04–7.11))	1.409 (1.27–2.18)	<0.0001
**MMP-7**	14.68 (13.71–17.78)	26.67 (24.71–58.26)	0.0037
**MMP-8**	216.7 (200.3–266.3)	42.63 (38.39–60.22)	<0.0001
**MMP-9**	646.4 (598.9–778.9)	32.39 (29.24–43.86)	<0.0001
**TIMP-1**	230.4 (216.1–264.7)	33.82 (29.27–47.45)	<0.0001
**TIMP-2**	291 (272.2–331.7)	50.77 (45.29–62.29)	<0.0001
**TIMP-3**	1.926 (2.44–5.95)	7.501 (7.09–8.25)	<0.0001
**TIMP-4**	11.01 (10.38–12.46)	5.027 (4.48–6.536)	<0.0001
**sCD14**	7.59 (7.42–10.69)	5.047 (4.382–7.11)	0.0106
**sCD163**	2.651 (2.41–4.03)	3.009 (2.54–5.44)	NS
**sPD-1**	8.202 (8.06–12.93)	3.758 (3.06–6.72)	<0.0001
**PDGF**	40.14 (42.04–72.48)	1.616 (1.44–3.07)	<0.0001

Values represent Geometric Means (+/−95% Confidence intervals). p values were calculated by the Mann-Whitney U test.

### Coincident helminth infection is associated with decreased systemic levels of acute phase proteins in active pulmonary tuberculosis

**Figure 1:**
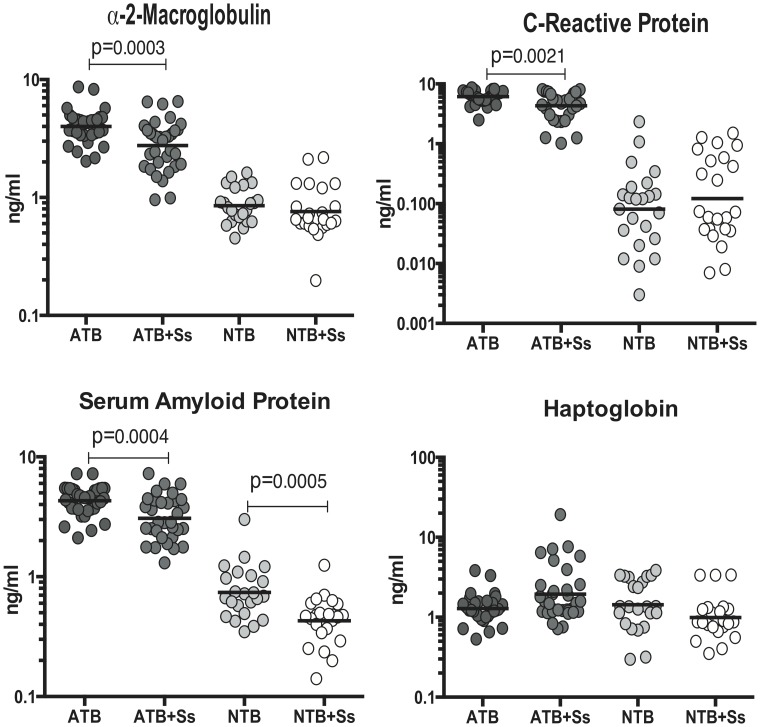
Helminth infections are associated with diminished plasma levels of acute phase proteins in active TB. The plasma levels of acute phase proteins - α-2M, CRP, SAA and Haptoglobin - were measured by multiplex ELISA in active pulmonary TB individuals with (ATB+Ss, n = 36) or without Strongyloides coinfection (ATB, n = 33) and in non-TB infected individuals with (NTB+Ss, n = 23) or without Strongyloides coinfection (NTB, n = 23). The results are shown as scatterplots with each circle representing a single individual and the bar representing the geometric mean. P values were calculated using the Kruskal-Wallis test with Dunn's multiple comparisons (* p<0.05, ** p<0.01, *** p<0.001, **** p<0.0001).

To determine the impact of Ss infection on the acute phase protein elevations seen in ATB, circulating levels of α-2M, CRP, SAA and haptoglobin in ATB and ATB+Ss individuals. As shown in Figure 1, infection with *S. stercoralis* in the context of active pulmonary TB was associated with significantly lower levels of α-2M (Geometric Mean of 4.0 ng/ml in ATB vs. 2.7 ng/ml in ATB+Ss), CRP (GM of 6.2 ng/ml in ATB vs. 4.3 ng/ml in ATB+Ss) and SAA (GM of 4.3 ng/ml in ATB vs. 3.1 ng/ml in ATB+Ss) - when compared to Ss-uninfected individuals with active TB. On the other hand, Ss infection was not associated with any significant alterations in the systemic levels of acute phase proteins (with the exception of SAA) in NTB individuals (Figure 1), indicating that helminth modulation of inflammatory markers is specific to active TB. Thus, Ss infection is associated with the dampening of systemic inflammation in active pulmonary TB.

### Coincident helminth infection is associated with decreased systemic levels of MMPs and TIMPs in active pulmonary tuberculosis

**Figure 2:**
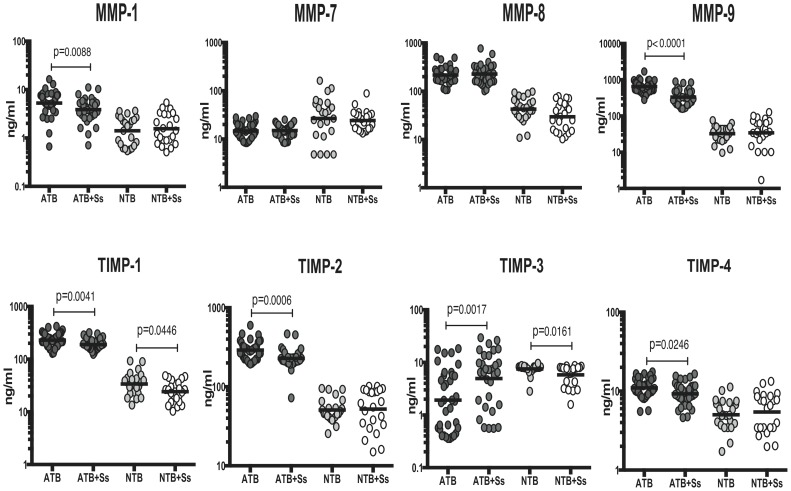
Helminth infections are associated with diminished plasma levels of MMPs and TIMPs in active TB. The plasma levels of MMPs (MMP-1, 7, 8 and 9) and TIMPs (TIMP-1, 2, 3 and 4) were measured by multiplex ELISA in active pulmonary TB individuals with (ATB+Ss, n = 36) or without Strongyloides coinfection (ATB, n = 33) and in non-TB infected individuals with (NTB+Ss, n = 23) or without Strongyloides coinfection (NTB, n = 23). The results are shown as scatterplots with each circle representing a single individual and the bar representing the geometric mean. P values were calculated using the Kruskal-Wallis test with Dunn's multiple comparisons (* p<0.05, ** p<0.01, *** p<0.001, **** p<0.0001).

To determine the impact of Ss infection on markers associated with tissue inflammation and remodeling at baseline (or steady state), we measured the circulating levels of MMP-1, 7, 8, 9 and TIMP-1, 2, 3, 4 in ATB and ATB+Ss individuals. As shown in Figure 2, infection with *S. stercoralis* in the context of active pulmonary TB was associated with significantly lower levels of MMP-1 (GM of 5.3 ng/ml in ATB vs. 3.9 ng/ml in ATB+Ss) and MMP-9 (GM of 646.4 ng/ml in ATB vs. 331.2 ng/ml in ATB+Ss) - when compared to Ss-uninfected individuals with active TB. Similarly, the plasma levels of TIMP-1 (GM of 230.4 ng/ml in ATB vs. 189.4 ng/ml in ATB+Ss), TIMP-2 (GM of 291 ng/ml in ATB vs. 228.6 ng/ml in ATB+Ss) and TIMP-4 (GM of 11.0 ng/ml in ATB vs. 9.3 ng/ml in ATB+Ss) were all significantly lower in ATB+Ss compared to ATB individuals (Figure 2). On the other hand, Ss infection was not associated with any significant alterations in the systemic levels of MMPs or TIMPs (with the exception of TIMP-1 and TIMP-3) in NTB individuals with or without Ss infection (Figure 2). Interestingly, TIMP-3 was the exception to both patterns with higher levels in ATB+Ss (compared to ATB) and lower levels in NTB+Ss (compared to NTB) individuals. Thus, Ss infection is associated with a TB specific modulation of circulating MMP and TIMP levels, indicating amelioration of disease severity in the context of helminth coinfection.

### Coincident helminth infection is associated with decreased systemic levels of immune activation markers in active pulmonary tuberculosis

**Figure 3:**
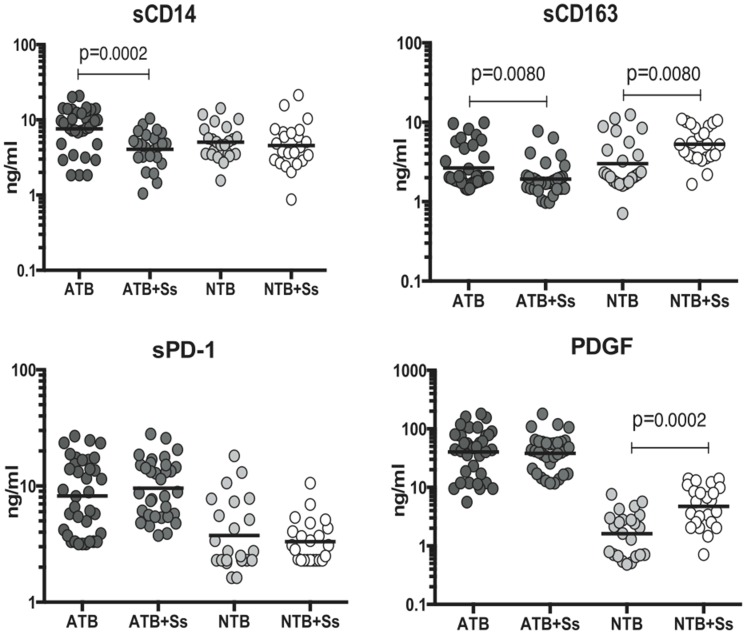
Helminth infections are associated with diminished plasma levels of sCD14 and sCD163 in active TB. The plasma levels of systemic immune activation markers (sCD14, sCD163, sPD-1 and PDGF) were measured by ELISA in active pulmonary TB individuals with (ATB+Ss, n = 36) or without Strongyloides coinfection (ATB, n = 33) and in non-TB infected individuals with (NTB+Ss, n = 23) or without Strongyloides coinfection (NTB, n = 23). The results are shown as scatterplots with each circle representing a single individual and the bar representing the geometric mean. P values were calculated using the Kruskal-Wallis test with Dunn's multiple comparisons (* p<0.05, ** p<0.01, *** p<0.001, **** p<0.0001).

To determine the impact of Ss infection on systemic immune activation markers at baseline (or steady state), we measured the circulating levels of sCD14, sCD163, sPD-1 and PDGF in ATB and ATB+Ss individuals. As shown in Figure 3, infection with *S. stercoralis* in the context of active pulmonary TB was associated with significantly lower levels of sCD14 (GM of 7.3 ng/ml in ATB vs. 4.1 ng/ml in ATB+Ss) and sCD163 (GM of 2.7 ng/ml in ATB vs. 1.9 ng/ml in ATB+Ss) - when compared to Ss-uninfected individuals with active TB. On the other hand, as shown in Figure 3, Ss infection was associated with significant elevations in the systemic levels of sCD163 and PDGF in NTB individuals. Thus, Ss infection is associated with the dampening of systemic inflammation in active pulmonary TB.

### Active TB associated elevation in the systemic levels of acute phase proteins, MMPs and TIMPs is independent of co-existent helminth infection

Since Ss infection was found to impart a profound effect on the systemic immune profile in active TB, we wanted to examine the impact of tuberculosis disease in helminth-infected individuals. To this end, we measured the circulating levels of all the above mentioned parameters, including acute phase proteins, MMPs, TIMPs and immune activation markers in ATB+Ss individuals and compared them to NTB+Ss individual to determine the contribution of Ss infection to changes in systemic markers in active TB versus controls. As shown in Table 3, active TB in the presence of Ss infection exhibited significantly higher levels of α-2m, CRP, SAA and haptoglobin in comparison to NTB individuals with Ss infection. Similarly, as shown in Table 3, active TB in the presence of Ss infection exhibited significantly higher levels of MMP -1, 8 and 9, TIMP - 1, 2 and 4 in comparison to NTB individuals with Ss infection. Finally, as shown in Table 3, active TB in the presence of Ss infection exhibited significantly higher levels of sPD-1 and PDGF in comparison to NTB individuals with Ss infection. Thus, active TB, independent of Ss co-infection, profoundly alters the circulating levels of inflammatory and immune activation markers.

**Table 3:** Systemic levels of immune parameters in active TB and TB uninfected individuals with concomitant Strongyloides infection.

	ATB+Ss	NTB+Ss	p value
**α-2-Macroglobulin**	2.763 (2.57–3.6)	0.7609 (0.65–1.08)	<0.0001
**C-Reactive Protein**	4.313 (4.13–5.61)	0.1215 (0.15–0.55)	<0.0001
**Serum Amyloid Protein**	3.069 (2.83–3.85)	0.4286 (0.37–0.56)	<0.0001
**Haptoglobin**	1.944 (1.57–4.06)	0.991 (0.81–1.59)	0.0004
**MMP-1**	3.846 (3.62–5.25)	1.551 (1.36–2.56)	<0.0001
**MMP-7**	14.97 (14.01–17.38)	24.22 (20.25–34.68)	0.0002
**MMP-8**	226.6 (204.4–302.2)	29.63 (26.55–46.42)	<0.0001
**MMP-9**	331.2 (304.1–445.7)	34.32 (33.55–62)	<0.0001
**TIMP-1**	189.4 (177.4–212.5)	24.18 (21.57–31.81)	<0.0001
**TIMP-2**	228.6 (213.1–263.6)	52.19 (47.91–74.16)	<0.0001
**TIMP-3**	4.935 (5.69–11.09)	5.808 (5.36–7.33)	NS
**TIMP-4**	9.256 (8.64–10.85)	5.448 (4.88–7.74)	0.0004
**sCD14**	4.059 (3.66–5.61)	4.559 (3.76–7.78)	NS
**sCD163**	1.924 (1.67–2.7)	5.295 (4.73–7.24)	<0.0001
**sPD-1**	9.575 (8.94–13.44)	3.32 (2.81–4.51)	<0.0001
**PDGF**	38.28 (35.76–61.36)	4.734 (4.39–8.13)	<0.0001

Values represent Geometric Means (+/−95% Confidence intervals). p values were calculated by the Mann-Whitney U test.

## Discussion

Epidemiological and clinical studies in humans as well as experimental studies in animal models strongly indicate that helminth infections can confer protection from a variety of inflammatory diseases such as allergy, autoimmunity and inflammatory bowel disease [8], [12]. The propensity of helminths to produce modulatory molecules to suppress anti-parasite and immuno-pathological responses at multiple levels renders them also with the ability to modulate host pathology during other chronic infections [8], [13]. Furthermore, although helminths are typically inducers of strong Th2 responses, they also induce regulatory T cells, alternatively activated macrophages and anti-inflammatory cytokines and antibodies to suppress host - protective (and possibly pathological) pro-inflammatory responses [6]. Thus, helminth products have been shown to modulate both Th1/Th17-mediated inflammation and Th2 dependent pathology [13]. Recent data suggest that parasitic worms can potentially provide benefits to humans in a clinical setting, and infection with helminths or their products have shown promise as potential therapeutics for inflammatory bowel disease and other inflammatory disorders [14], [15]. We and others have previously shown that helminth infections can modulate both the innate and adaptive arms of the immune system in active and latent TB [10]. In this study, we sought to elucidate the modulatory function (if any) of a chronic helminth infection on the systemic pathological responses that characterize disease activity and severity in pulmonary TB. *S. stercoralis* infection is known to overlap geographically with *M. tuberculosis* [16] and, more significantly, Ss infection in the mouse has been shown to impair immune responses to TB infection [9]. Therefore, we elected to examine the interaction of Ss and *M. tuberculosis* at the systemic level.

Acute phase proteins are non-specific serum proteins that are elevated in patients with TB [17]. Recently, CRP has been proposed as a candidate biomarker for active infection with Mtb [18]. Point-of-care CRP testing has been shown to be of use in the clinical evaluation of respiratory tract infections in adults and of fever in children [19]. In addition, previous studies also report that SAA is another important candidate biomarker for TB [20]. Our study reveals clearly reveals that in addition to CRP and SAA, α-2M is also a remarkably good biomarker in distinguishing TB from non-TB individuals. More importantly, however, our data also show that all three markers of systemic inflammation in TB are significantly modulated by the presence of coincidental Ss infection and that this modulation is relatively TB disease specific. While acute phase proteins are typically (but not always) markers of acute inflammation, systemic immune activation markers more accurately reflect inflammatory pathology in chronic infections. sCD14 and sCD163 are markers of monocyte/macrophage activation and their levels in the blood usually reflect chronic immune activation involving myeloid cells [21]. In addition, sCD163 and sCD14 have been shown previously to server as plasma markers of active TB [22], [23]. PDGF is a pro-fibrotic growth factor that has been directly linked to increased fibrosis in TB patients [24]. Finally, we also examined the circulating levels of sPD-1, which is a soluble form of the T cell co-receptor PD-1 and has been implicated in disease activity in various inflammatory conditions [25], [26]. Our data first confirms the utility of these molecules (with the exception of sCD163) as putative biomarkers of active TB. More interestingly, our data reveal a profound impact on Ss co-infection on the systemic levels of some of these biomarkers, especially the ones associated with monocyte/macrophage activation. Helminth infections are known to have a major impact on the function of antigen-presenting cells, including dendritic cells, monocytes and macrophages [6]. Hence, it is not surprising to find an important effect of Ss infection on monocyte activation markers in active TB.

MMPs and TIMPs are important additional factors in the pathogenesis of TB due to their ability drive immune-mediated pathology [27], [28]. MMPs are zinc dependent proteases, associated with breakdown of the extracellular matrix and tissue remodeling [29], [30]. TIMPs are specific inhibitors of MMPs and help control tissue pathology [29], [30]. Thus, various MMPs have been shown to be upregulated in peripheral blood and at the site of disease in TB infection and their circulating levels have been shown to accurately reflect lung pathology and TB disease severity [27]. This is because MMPs play a major role in the underlying mechanism of lung extracellular matrix destruction in TB owing to their unique ability to degrade fibrillar collagens and other matrix components [28]. This matrix destruction is central to the development of lung cavitation and necrosis, which in turn is the mainstay of TB transmission [31]. Very few studies have examined the expression of TIMP in TB infections. While TIMPs are clearly known to bind and inhibit the function of MMPs, it is also becoming evident that TIMP binding to MMP can enhance the activity of certain MMPs [30]. Moreover, TIMPs appear to also exert MMP independent activities in tissue remodeling [32]. Our data clearly reveal two very interesting features concerning the family of tissue remodeling enzymes: (i) systemic MMPs and TIMPs appear to be very good biomarkers in distinguishing TB from non-TB individuals and (ii) the concentrations of MMPs and TIMPs are downregulated by the presence of Ss co-infection. Helminth infections are known to modulate the expression pattern of MMPs and TIMPs [33] and in the case of certain helminth infections can act of inducers of these factors themselves [34]. Our data, however, suggest that Ss infection in the context of TB disease plays a modulatory role with respect to the production of pro-fibrotic factors and hence can potentially impact the degree of lung pathology. Unfortunately, we were unable to systematically collect radiological information on the extent of pulmonary disease in this study, and therefore we are unable to corroborate the systemic findings at the pulmonary level.

It has been speculated that there are two evolutionarily conserved defense strategies against infection that limit host disease severity [35], [36]. The first is dependent on the capacity of the host's immune system to reduce pathogen burden. In our study, since we did not observe any significant differences in the sputum smear grades between the two groups, we conclude that the bacterial load was not affected by the presence of coincidental Ss infection. The second defense strategy is commonly known as “disease tolerance” and is thought to affect the fitness cost of infection i.e. metabolic or other pathways that limit disease severity [35]. While we have not directly identified the mechanism by which Ss infection affects disease severity in TB, it is tempting to speculate that both alteration in the host immune and non-immune defense pathways are being modulated by co-infection. It is likely that the regulatory pathways induced during chronic helminth infections play a role in restoring homeostasis and normal tissue function and in promoting wound healing/repair and anti-inflammatory responses [37]. One of the major problems in terms of current TB research and clinical demands is the increasing number of cases of extensively drug resistant and treatment refractory TB [38]. To combat this problem, a great deal of emphasis is now laid on host-directed therapies targeting inflammatory processes that can be deleterious and lead to immune exhaustion in TB [39]. Candidates for such interventions may be biological agents or already approved drugs repurposed to interfere with inflammatory processes [40]. Helminth immuno-modulators, if approved, could feasibly serve as another approach to tackle this issue.

Our findings have important implications for the design of studies investigating immunologically-based biomarkers to distinguish active from latent TB and to monitor response to therapy. Patients from helminth-endemic regions have previously been reported to have less extensive disease compared to those from helminth-free regions [41], but it is not clear whether this is due to ethnic differences, to underlying host - pathogen differences, or to access to therapy. Our data add another layer of complexity to this conundrum and suggest that the presence of a different chronic infection could also have an effect on the disease manifestations in TB. While our study is clearly preliminary and needs to be confirmed in a much larger setting – and while longitudinal studies examining the effect of anti-helminth treatment on TB pathology needs to be examined – our data clearly illustrate the powerful regulatory effects that helminth infections can exert on the immune response to third party infections. A more detailed examination of the regulatory pathways influencing this effect of helminth infection on TB could provide clues to unravel potentially beneficial avenues to combat this pervasive infection and disease.

## Supporting Information

Figure S1 Strobe checklist.
(DOC)
